# Physical and biological properties of electrospun poly(d,l‐lactide)/nanoclay and poly(d,l‐lactide)/nanosilica nanofibrous scaffold for bone tissue engineering

**DOI:** 10.1002/jbm.a.37199

**Published:** 2021-05-04

**Authors:** Francesco Lopresti, Francesco Carfì Pavia, Manuela Ceraulo, Elisa Capuana, Valerio Brucato, Giulio Ghersi, Luigi Botta, Vincenzo La Carrubba

**Affiliations:** ^1^ Department of Engineering University of Palermo, RU INSTM Palermo Italy; ^2^ Department of Biological, Chemical and Pharmaceutical Sciences and Technologies University of Palermo Palermo Italy; ^3^ ATeN Center University of Palermo Palermo Italy

**Keywords:** electrospinning, nanoclay, nanosilica, polylactic acid, pre‐osteoblastic cells, tissue engineering

## Abstract

Electrospun scaffolds exhibiting high physical performances with the ability to support cell attachment and proliferation are attracting more and more scientific interest for tissue engineering applications. The inclusion of inorganic nanoparticles such as nanosilica and nanoclay into electrospun biopolymeric matrices can meet these challenging requirements. The silica and clay incorporation into polymeric nanofibers has been reported to enhance and improve the mechanical properties as well as the osteogenic properties of the scaffolds. In this work, for the first time, the physical and biological properties of polylactic acid (PLA) electrospun mats filled with different concentrations of nanosilica and nanoclay were evaluated and compared. The inclusion of the particles was evaluated through morphological investigations and Fourier transform infrared spectroscopy. The morphology of nanofibers was differently affected by the amount and kind of fillers and it was correlated to the viscosity of the polymeric suspensions. The wettability of the scaffolds, evaluated through wet contact angle measurements, slightly increased for both the nanocomposites. The crystallinity of the systems was investigated by differential scanning calorimetry highlighting the nucleating action of both nanosilica and nanoclay on PLA. Scaffolds were mechanically characterized with tensile tests to evaluate the reinforcing action of the fillers. Finally, cell culture assays with pre‐osteoblastic cells were conducted on a selected composite scaffold in order to compare the cell proliferation and morphology with that of neat PLA scaffolds. Based on the results, we can convince that nanosilica and nanoclay can be both considered great potential fillers for electrospun systems engineered for bone tissue regeneration.

## 1 INTRODUCTION

Over the recent years, biopolymers find a wide range of advanced applications that include biodegradable food packaging,^1^, ^2^ bioremediation,^3^ biosensing,^4^ controlled drug release,^5^ and tissue engineering.^6^ In this context, there is a growing interest in functional biopolymeric porous scaffolds exhibiting high mechanical properties with the ability to support cell attachment and proliferation.^7^, ^8^, ^9^ Electrospinning is becoming more and more attractive for its simplicity and flexibility if compared with the other scaffold fabrication techniques.^10^, ^11^ In fact, the electrospinning technique permits easy control of the fiber diameter, porosity, and mechanical properties of the final scaffolds by changing the processing parameters and the materials used.^12^, ^13^, ^14^ Electrospun membranes are often engineered to be involved in advanced applications including catalysis,^15^, ^16^ controlled drug release,^17^, ^18^ bioprocess intensification,^12^, ^19^ biosensing,^20^ food packaging,^21^ but a still‐open challenge is the preparation of mats with adequate properties for tissue engineering purposes.^22^ Beyond its versatility in material selection, which can include both natural and synthetic polymer, electrospinning also provides the possibility to include nanoparticles into the polymeric fibers.^23^ By considering the unique properties related to nanometric size and high specific surface, the incorporation of functional nanoparticles into an electrospun polymer matrix can provide substantial properties enhancements, even at low nanoparticles content.^24^, ^25^

This feature can be used to modify specific properties of nanofibrous polymers by increasing their mechanical strength,^26^ their bioactivity,^27^ and/or endowing them with additional features including electrical conductivity.^28^ The chemical composition and the particle dimension strongly affect the matrix/particles interaction and, as a consequence, the final properties of polymer‐based composites.^29^, ^30^ For this reason, several inorganic nano‐sized particles, such as metals, carbon‐based materials, silica, and clays have been extensively studied in the field of polymer‐based nanocomposites in order to enhance their performances for specific applications such as environmental remediation, electromagnetic interference shielding, sensing,^31^ supercapacitors,^32^ packaging, automotive, and solar energy fields.^25^

In tissue engineering applications, various types of inorganic nanofiller such as carbon nanotubes,^33^ graphene,^26^ and hydroxyapatite^27^ have been used to produce polymer/inorganic biocomposite fibers, leading to high‐performance nanofibrous mats. In this context, nanosilica and nanoclay are one of the most investigated nanofillers for polymer matrix due to several interesting properties including low thermal conductivity, chemically inertness, and non‐toxicity^34^, ^35^, ^36^, ^37^, ^38^, ^39^, ^40^, ^41^, ^42^, ^43^, ^44^, ^45^ that can be exploited for tissue engineering purposes and other biomedical applications.^46^, ^47^, ^48^, ^49^, ^50^, ^51^, ^52^, ^53^, ^54^, ^55^

Although several research articles report the physical characterization of non‐bioresorbable polymer/silica and polymer/clay composite electrospun fibers,^36^, ^37^, ^39^, ^40^, ^41^, ^42^, ^43^, ^44^, ^45^, ^56^ relatively a few articles deal with biopolymers‐based electrospun nanocomposites^46^, ^47^, ^48^, ^49^, ^50^, ^51^, ^57^ and even less with their interaction with living cells for tissue engineering applications.^52^, ^53^, ^54^, ^55^, ^58^

Singh et al. developed multifunctional mesoporous silica‐shelled polycaprolactone hybrid nanofiber scaffolds for bone regeneration exhibiting excellent mechanical functionality.^52^ Mehrasa et al. prepared aligned nanofibrous composites made of poly(lactide‐co‐glycolide)/gelatin/mesoporous silica by electrospinning for nerve tissue engineering applications.^53^ Koosha et al. produced chitosan/polyvinyl alcohol/montmorillonite (MMT) nanofibrous scaffolds highlighting that the mechanical properties of the nanocomposite were highly improved by the addition of only 1 and 3 wt% of MMT. Furthermore, these nanocomposite scaffolds were found to be biocompatible showing no adverse cytotoxic effect on human fibroblast cells.^54^ Gaharwar et al. fabricated nanoclay‐enriched poly(ε‐caprolactone) electrospun scaffolds for osteogenic differentiation of human mesenchymal stem cells (hMSCs) able to enhance the attachment, proliferation, and differentiation of hMSCs if compared to the neat PCL electrospun scaffolds.^55^

To the best of our knowledge, there is not any available work focusing on the direct comparison of polylactic acid (PLA)‐based electrospun scaffold containing either silica (AS) or clay (CLO) nanoparticles for potential tissue engineering purposes. PLA is a commonly used material for tissue engineering applications due, in part, to its ability to degrade into the naturally occurring lactic acid under physiological conditions. Other exceptional features are the low immunogenicity and the interesting mechanical properties.^59^, ^60^ However, PLA has some drawbacks such as biological inertness and low cell adhesion that need to be addressed.^61^

Therefore, this work aim is to compare the physical properties and the in vitro cytotoxicity and cell attachment of pre‐osteoblastic cells on PLA/AS and PLA/CLO electrospun scaffolds. The inclusion of the nanoparticles into the electrospun mats was investigated with Fourier transform infrared spectroscopy in attenuated total reflectance (FTIR‐ATR). The rheological measurements conducted on the polymeric solution/suspensions were correlated with the fiber morphology that was analyzed through scanning electron microscopy (SEM) and image processing. Uniaxial tensile tests were carried out in order to evaluate the reinforcing effect of both AS and CLO for three different compositions (1, 3, and 5 wt%). Differential scanning calorimetry (DSC) permitted the investigation of the crystallinity of the composites. Finally, pre‐osteoblastic cells were seeded on the composite scaffolds in order to compare their vitality and morphology on the PLA/AS and PLA/CLO bionanocomposites with that of neat PLA scaffolds.

## 2 MATERIALS AND METHODS

### 2.1 Materials

PLA (2002D, NatureWorks) was used in this work. Acetone (Ac) and chloroforms (TCM) were purchased from Sigma–Aldrich. The commercial nanosilica is Aerosil R812 fumed silica supplied by Evonik Industries AG (Evonik Degussa) with a declared specific surface of 230–290 m^2^/g, modified with hexamethyldisilazane. The commercial clay is Cloisite 30B, supplied by Southern Clay Products. The clay is a MMT modified by 90 meq/100 g of bis(2‐hydroxyethyl)methyl tallow alkylammonium cations. Before processing, the polymer and the fillers were dried overnight at 90°C under vacuum in order to avoid PLA hydrolytic degradation during processing.^62^ All the reactants were ACS grade (purity >99%).

### 2.2 Electrospinning processing

PLA nanofibers, PLA/AS, and PLA/CLO electrospun composites preparation followed a fabrication route similar to those described in a previous work.^27^ In brief, PLA (10 wt%) was dissolved in TCM:Ac (2:1 vol) at room temperature under continuous magnetic stirring overnight. PLA/AS and PLA/CLO suspensions were prepared by adding AS or CLO particles to the solvent system that was then subjected to ultrasonication for a total of 1 hr, and finally adding the PLA with a weight concentration of 10 wt% with respect to TCM:Ac mixture. AS and CLO were added in the solvent system in order to achieve 1, 3, and 5 wt% with respect to the polymer phase, according to scientific literature.^54^

The PLA, PLA/CLO, and PLA/AS scaffolds were prepared by using semi‐industrial electrospinning equipment (NF‐103, MECC CO., LTD., Japan) equipped with a cylindrical grounded rotary drum (diameter = 10 cm). The polymeric solution/suspensions were filled to a 5 ml syringe fitted with 19‐gauge stainless steel. The following constant parameters were set: flow rate, 1 ml/hr; needle tip‐collector distance, 13 cm; high voltage, 15 kV; temperature, 25°C, collector angular speed, 10 rpm; processing time, 120 min; According to the above‐mentioned parameters, approximately 70 μm thick membranes were obtained. In order to remove any residual solvents, the collected scaffolds were dried for 48 hr under fume hood.

### 2.3 Morphological analysis

The morphology of the scaffolds was evaluated by scanning electron microscopy, (Phenom ProX, Phenom‐World) and by transmission electron microscopy by using the Versa 3D Dual Beam Scanning Electronic Microscope (Thermo Fisher, FEI) equipped with a retractable STEM detector. For SEM analysis, circular samples (diameter = 10 mm) were attached by using adhesive carbon tape on the aluminum stub. Before the analysis, the samples were sputter‐coated with gold for 60 s under argon atmosphere by using a Sputtering Scancoat Six (Edwards) in order to avoid electrostatic discharge during the test.^63^ The SEM was set with an accelerated voltage equal to 10 kV. The samples for TEM investigation were prepared by the direct deposition of the electrospun nanofibers onto the carbon‐coated copper grid. The samples were analyzed by using an accelerated voltage equal to 30 kV.

### 2.4 CLO and AS specific surface

Surface area measurements were performed by using an autosorb iQ‐MP/XR (Quantachrome) instrument. Before measurement, each sample was outgassed under vacuum at 120°C for 3 hr. The surface area was determined by physical adsorption of N_2_ at the liquid nitrogen temperature, using the Brunauer–Emmett–Teller (BET) equation.^64^

### 2.5 FTIR/ATR analysis

FTIR/ATR analysis (Perkin‐Elmer FT‐IR/NIR Spectrum 400 spectrophotometer) was carried out in order to investigate the chemical surface properties of the samples were assessed by spectroscopic analysis. Four accumulations scans with a resolution of 4 cm^−1^ were collected for each sample in the range 4,000–400 cm^−1^.

### 2.6 Particles size and fiber diameter distributions

A dedicated image processing software was used to investigate the fiber diameter and the particle size distribution of the electrospun mats. ImageJ on SEM images of AS and CLO particles was used to determine the particle size distribution while a plugin for ImageJ (DiameterJ) was used to investigate the fiber diameter distribution.^65^

### 2.7 Differential scanning calorimetry

DSC (Setaram, model DSC131) was used to investigate the calorimetric properties of the scaffolds. The analysis was carried out with two cycles of heating from room temperature to 190°C at 10°C/min heating rate under nitrogen flow on electrospun samples with approximately the same weight (~ 5 mg) sealed in aluminum pans.

PLA and PLA‐based composites crystallinity degree (χ) were calculated according to the following equation^66^:

(1) $$\chi \;\left(\%\right)=\frac{∆H_{m}-∆H_{\mathrm{cc}}}{∆{H^{0}}_{\mathrm{PLA}}\times X_{\mathrm{PLA}}}\times 100$$

where Δ*H*_*cc*_ and Δ*H*_*m*_ are the cold crystallization and melting enthalpy of the samples, respectively. *X*_PLA_ is the weight fraction of PLA and Δ*H* ^0^_*m*_ is the melting enthalpy of 100% crystalline PLA equal to 93.7 J/g.^66^

### 2.8 Polymeric solutions/suspensions complex viscosity

A plate–plate rotational rheometer Mars (Thermofisher Rheological) with 25 mm parallel‐plate geometry at 25°C was used to perform the characterization of the polymeric solutions/suspensions complex viscosity. Oscillatory frequency sweep tests were performed at a constant stress of 1 Pa with an increase of angular frequency from 1 to 100 rad/s.

### 2.9 Water contact angle measurements

FTA 1000 (First Ten Ångstroms, UK) instrument was used to perform the static contact angles measured by using distilled water (DW) as fluids. In particular, a droplet of DW (~ 4 μl) was dropped on the scaffold and the images were taken after 10 s from the DW deposition. At least seven spots of each composite nanofiber mat were tested and the average value was taken.

### 2.10 Mechanical properties

A laboratory dynamometer (Instron model 3365) equipped with a 1 kN load cell was used to perform the tensile mechanical measurements on rectangular‐shaped specimens (10 × 90 mm).

The electrospun mats were cut off from along the radial direction of the cylindrical collector. Due to the high elongation of the samples, the tensile tests were carried by using a double crosshead speed: 1 mm/min up to 10% strain and 50 mm/min until fracture. The initial length of the samples was 20 mm while the thickness of each sample was measured before the test. From the nominal stress–strain curves, the following mechanical parameters were obtained: elastic modulus (E), tensile strength (TS), and deformation at break (ε_b_). Seven samples were tested for each material and the average values of the mechanical parameters were reported with their standard deviations.

### 2.11 In vitro cell cultures

For biological tests, pre‐osteoblastic MC3T3‐E1 cells (Sigma–Aldrich) were utilized. Cells were grown in Dulbecco's modified Eagle's medium (Sigma–Aldrich) supplemented with 10% of fetal bovine serum, 1% of glutamine, and 1% of streptomycin/penicillin at 37°C with 5% CO_2_. Pure PLA, PLA/AS 1%, PLA/AS 5%, PLA/CLO 1%, and A PLA/CLO 5% scaffolds were cut to square‐shaped samples, with a surface of about 1 cm^2^. The scaffolds were then sterilized under UV for 2 hr and soaked for 12 hr in a complete culture medium. Twenty microliters of cellular suspension were inoculated onto each scaffold in order to reach a seeding concentration of 5 × 10^4^ cells/cm^2^. After 90 min of incubation at 37°C and 5% CO_2_ (to promote cell adhesion), each scaffold was transferred into a medium‐filled well of a 24 multiwell plate.

### 2.12 Cell viability assay

AlamarBlue*™* Cell Viability Reagent (Invitrogen) was used to evaluate cell proliferation. The samples were transferred into clean wells and each scaffold was incubated at 37°C and 5% CO_2_ for 3 hr with 500 μl of an Alamar Blue reagent (10×) diluted (1:10) in Medium. The fluorescence values were read on a plate reader; excitation wavelength was 530/25 (peak excitation is 570 nm) whereas emission wavelength was 590/35 (peak of emission is 585 nm). The number of living cells is directly proportional to the fluorescence value. The assays were carried out at 0, 4, 7, and 11 days of culture in triplicate for each time. Scaffolds without cells were used as blank for each measurement.

### 2.13 Analysis of the cell morphology on the surface of the scaffolds

MC3T3‐E1 cells adhered onto scaffolds were observed through SEM. Samples were extracted from the wells, rinsed with D‐PBS pH 7.4, and fixed with glutaraldehyde 4% (vol/vol) at 4°C for 2 hr. After fixation, samples were abundantly washed with deionized water and dehydrated with increasing ethanol series (25, 50, 75, and 100% vol/vol). Finally, samples were dried, gold‐sputtered, and observed with a SEM‐FEI QUANTA 200F microscope (Thermo Fischer).

### 2.14 Statistical analysis

Statistical analyses of the data were performed through one‐way analysis of variance, and when applicable, data were compared using the Student's *t* test. *p*‐value <.05 was considered statistically significant.

## 3 RESULTS

### 3.1 Morphology and surface chemistry of the electrospun mats and suspensions viscosity

**FIGURE 1:**
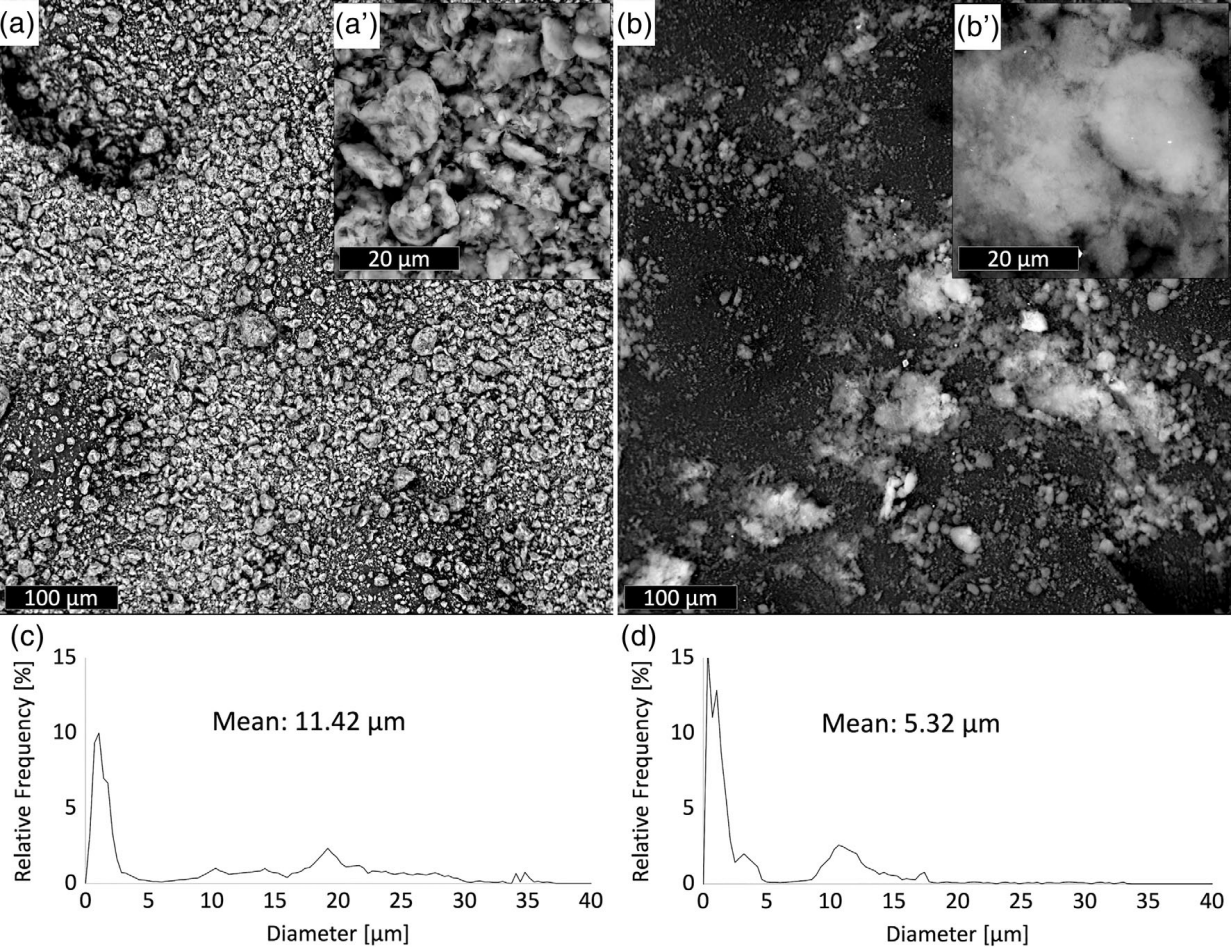
Scanning electron microscopy (SEM) micrographs of (a) clay (CLO) and (b) silica (AS) particles. Particle size distribution of (c) CLO and (d) AS particles. Characterizations of both particles were carried out after 1 hr sonication in TCM:Ac (2:1 vol)

Figure 1a,b shows the morphology of CLO and AS, respectively, after 1 hr of sonication in TCM:Ac 2:1 vol in order to observe their morphology after the same treatment used for the preparation of the PLA/particles suspensions. The morphology of CLO is characterized by aggregates presenting a spread particle size distribution with a mean diameter of 11.42 μm. In particular, the particle size distribution (Figure 1c) shows a high frequency of particles around 1 μm and several aggregates presenting a diameter ranging from 10 to 50 μm (not in scale of the graph for the sake of clarity). As evident in the inset of Figure 1a, CLO aggregates were found to be remarkably packed, surrounded by smaller particles. Differently, the shape of AS aggregates appeared less packed if compared with that of CLO (inset in Figure 1b') exhibiting a mean diameter of 5.32 μm. The size of AS aggregates never exceeded 17 μm with a peak of distribution at 350 nm (Figure 1d).

**FIGURE 2:**
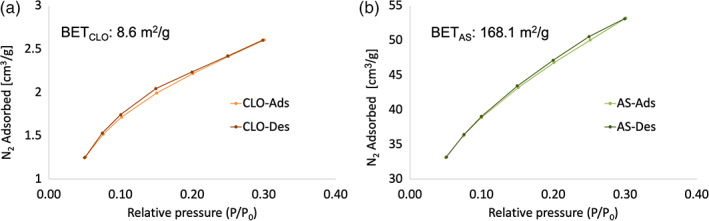
N_2_ adsorption–desorption isotherms of (a) clay (CLO) and (b) silica (AS) nanoparticles

The specific surface areas of AS and CLO were measured by nitrogen adsorption/desorption analysis following the BET and the results are presented in Figure 2. The specific surfaces are 168.1 and 8.6 m^2^/g for AS and CLO, respectively, thus confirming the low dimensions of AS particles.

**FIGURE 3:**
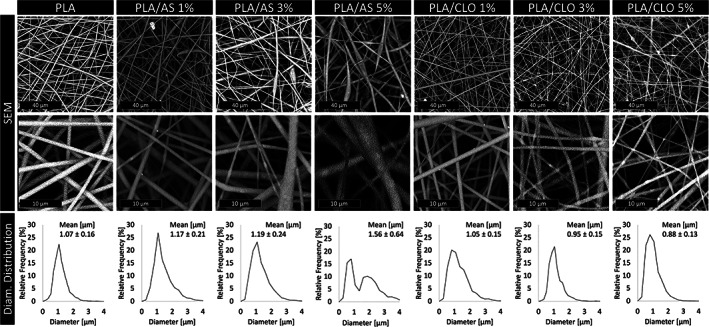
Scanning electron microscopy (SEM) micrographs of polylactic acid (PLA), PLA/silica (AS), and PLA/clay (CLO) electrospun nanocomposites mats at different AS or CLO concentrations and their corresponding fiber diameter distribution

SEM micrographs of electrospun PLA and its composite are presented in Figure 3 as well as their corresponding diameter distribution. The micrograph of PLA fibers shows the typical morphology of an electrospun material with smooth and randomly oriented fibers in the nanoscale (PLA_mean diameter_ = 1.07 ± 0.16 μm). PLA fibers displayed a rather homogeneous diameter as confirmed by the narrow peak of the fiber diameter distribution. All PLA/AS composites produced in this work exhibited higher mean fiber diameter and more spread peak of the fiber diameter distribution than neat electrospun PLA. Specifically, the mean fiber diameter of PLA/AS 1% and 3% were approximately 10% higher than PLA fibers while PLA/AS 5% mean fiber diameter was even 56% higher than PLA. SEM images clearly reveal that PLA/AS fibers diameter were not homogeneous but characterized by thin fibers that enlarge in bead‐like structures. The fiber surface is smooth, and it is possible to observe some bright AS particles on the surface of the high diameter portion of PLA/AS fibers and inside their small‐diameter portion. As expected, the number of visible AS particles, both on the surface and inside the fibers, increased upon increasing their concentration in the polymeric solution, corroborating the successful loading control of AS. The increase of the PLA/AS fiber diameter could be ascribed to the presence of nanosilica aggregates, but, overall, it is possible to observe a good dispersion of AS particles. On the other hand, all PLA/CLO fibers showed a lower mean fiber diameter than neat electrospun PLA as already observed for different electrospun matrices filled with clays.^54^, ^57^ More in detail, PLA/CLO 1, 3, and 5% mean fiber diameters were approximately 2, 11, and 18% lower than PLA fibers, respectively. Furthermore, the fiber diameter distribution peaks of PLA/CLO systems were narrower than that of PLA fibers, as quantitatively described by the lower standard deviation evaluated from the graphs shown in Figure 2. CLO particles are brighter than PLA thus easily visible also when they are embedded in the fibers. Interestingly, only some CLO aggregates around 5 μm are visible in PLA/CLO 5% that is much lower than that observed for the sonicated CLO particles. About the filler dispersion, from SEM images it can be noticed that PLA/AS nanocomposites displayed a higher concentration of particles in the bead‐like regions of the fibers, in particular at the higher AS concentrations.

**FIGURE 4:**
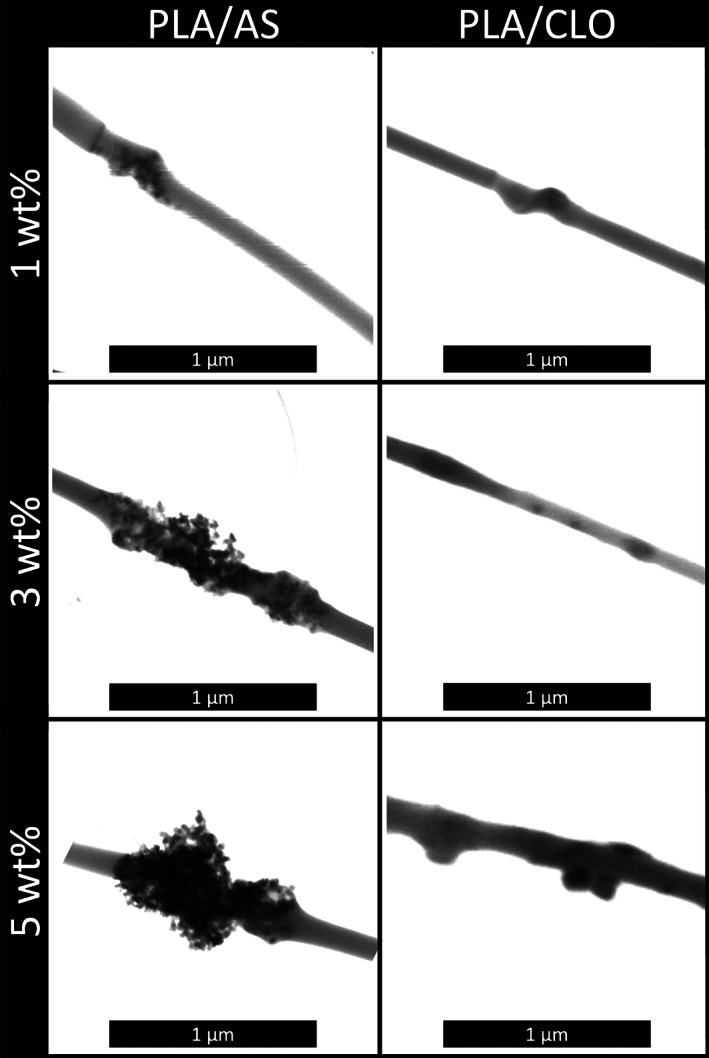
STEM micrographs of polylactic acid/silica (PLA/AS) and PLA/clay (CLO) electrospun nanocomposites mats at different AS or CLO concentrations

Figure 4 shows the STEM images of the electrospun PLA/CLO and PLA/AS nanofibers. Among the electrospun mat, fibers with diameters about 100 nm enabled direct imaging of the interior morphology of the composite fibers by STEM investigation. Although STEM was performable only on the thinner fibers of the mats, it permitted us to obtain information that helps to further explain SEM observations. More in detail, for the PLA/CLO mats, a large percentage of stacked montmorillonite is observed rather than individual layers. The number and size of CLO stacks increased upon increasing the CLO wt% filled into the polymer matrix. Similarly, with the increase in silica content, the PLA/AS nanofibers presented greater aggregation or agglomeration of silica nanoparticles. At higher silica contents (i.e., 3 and 5 wt%), the dispersibility of silica in PLA fibers seems to be much difficult, which may contribute to the final irregular surface morphology of nanofibers as already observed in other polymer/silica electrospun systems.^41^

**FIGURE 5:**
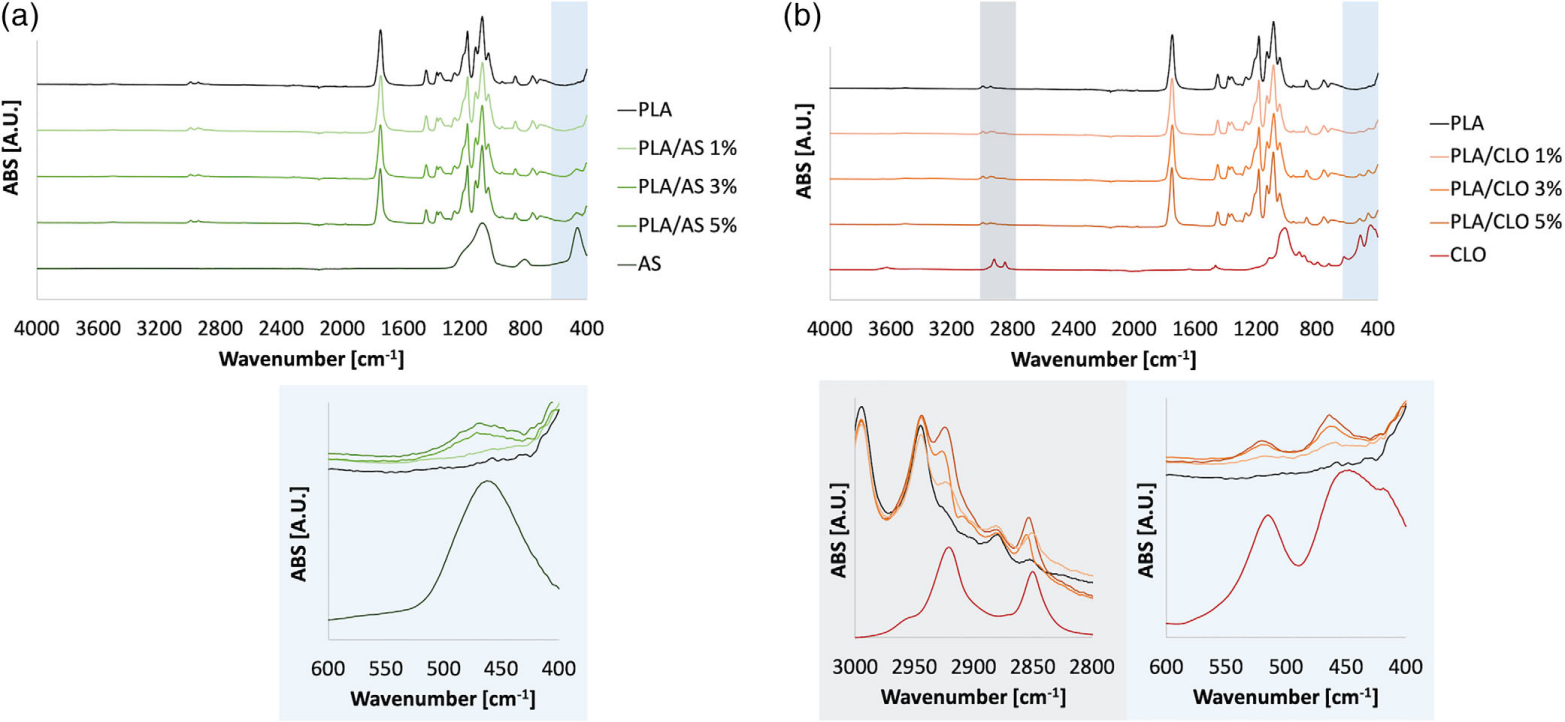
Fourier transform infrared spectroscopy in attenuated total reflectance (ATR‐FTIR) carried out on (a) silica (AS), polylactic acid (PLA) and PLA/AS electrospun nanocomposites and (b) clay (CLO), PLA and PLA/CLO electrospun nanocomposites for different filler concentration

The ATR‐FTIR spectrum of PLA (Figure 5a,b) showed several peaks usually attributed to this polymer such as the carbonyl stretch at 1747 cm^−1^, the C–O stretch at 1180, 1,129, and 1,083 cm^−1^, as well as the OH bend at 1044 cm^−1^.^67^ ATR‐FTIR spectra carried out on neat AS and CLO powders are shown in Figure 5a,b respectively. The FTIR‐ATR absorbance spectra of AS nanoparticles showed three main distinguishable peaks at 1,103, 805, and 471 cm^−1^ related to asymmetric Si–O–Si stretching, SiO_4_ tetrahedron ring, and O–Si–O deformation, respectively.^68^ PLA/AS electrospun nanocomposites spectra were characterized by the same spectrum of PLA plus a peak at 471 cm^−1^ (O–Si–O deformation of AS) that increases upon increasing the AS concentration in the polymer. The FTIR spectra of neat Cloisite® 30B and PLA/CLO electrospun nanocomposites are shown in Figure 5b. Cloisite® 30B reveals absorption bands at 3631 cm^−1^ representing the Si–OH stretching band, at 1048 cm^−1^ representing the stretching vibration of Si–O–Si from silicate, and at 918 cm^−1^ due to Al–OH–Al deformation of aluminates.^69^ The bands located at 2,925, 2,853, and 1,470 cm^−1^ are due to the organic modification, which is assigned to the CH vibrations of methylene groups (asymmetric stretching, symmetric stretching, and bending, respectively).^69^ Bands at 530 and 470 cm^−1^ are assigned to the stretching modes of Al–O and Mg–O, respectively.^70^

The FTIR spectra of PLA/CLO electrospun nanocomposites show the characteristic peak of neat PLA plus peaks that increased upon increasing the filler content in the nanocomposites at 2925, 2,853 cm^−1^ due to alkylammonium ions in Cloisite 30B and at 530 and 470 cm^−1^ due to Al–O and Mg–O, respectively.

**FIGURE 6:**
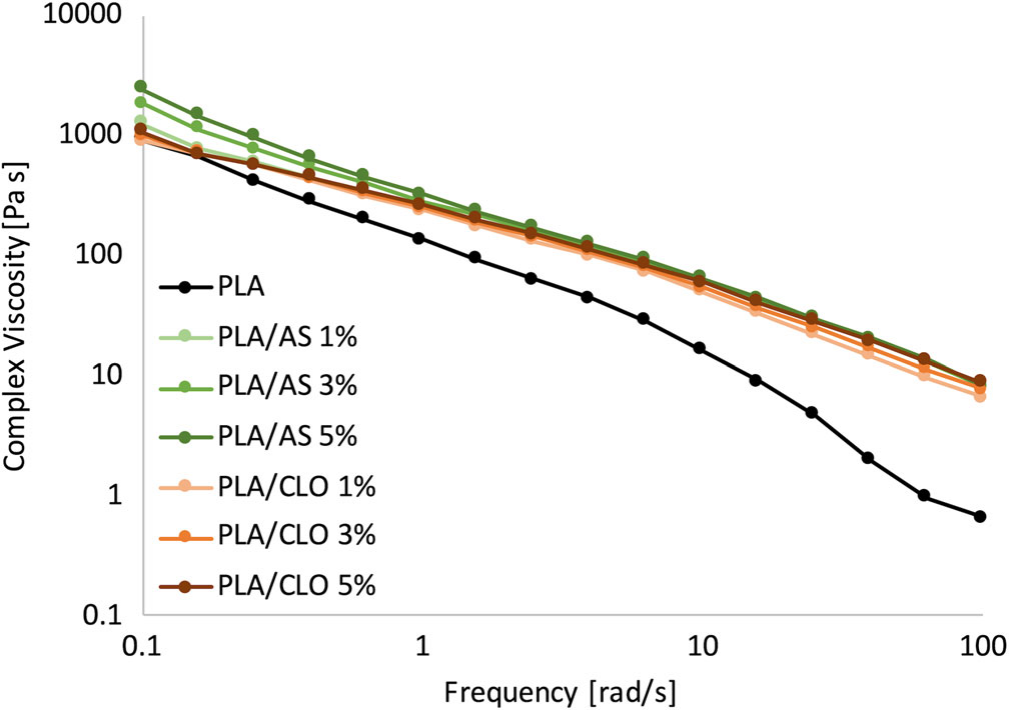
Complex viscosity of the polylactic acid (PLA) solution and PLA/silica (AS) and PLA/clay (CLO) suspensions

It is well known that that electrospun fiber diameter is strongly affected by solution viscosity.^26^, ^71^ The complex viscosity of PLA solution, as well as PLA/AS and PLA/CLO suspensions, were measured in order to evaluate the correlation between the viscosity and the fiber diameter of electrospun mats, as a function of frequency (Figure 6). As expected, if compared with neat PLA solution, all the PLA/AS and PLA/CLO suspensions showed higher viscosity in particular at the higher frequency, similarly to what was already reported in a previous work.^27^ More in detail, PLA/AS suspensions viscosity was higher than that of PLA and PLA/CLO also at low frequency, and it increased upon increasing the AS content.

The effect of the concentration of AS on the viscosity of PLA/AS suspensions can be observed only at the lowest frequencies while all the PLA/AS curves are overlapped from ~2.5 rad/s until the end of the test, remaining much higher than that of PLA solutions. Starting from ~2.5 rad/s also PLA/AS and PLA/CLO viscosity overlapped remaining almost similar to each other up to 100 rad/s. Small differences at the highest frequencies can be observed between PLA/AS and PLA/CLO suspensions since the latter at 1 and 3% concentration of CLO showed a slightly lower viscosity if compared to the other systems but still much higher than that of PLA solution.

### 3.2 Wettability

**FIGURE 7:**
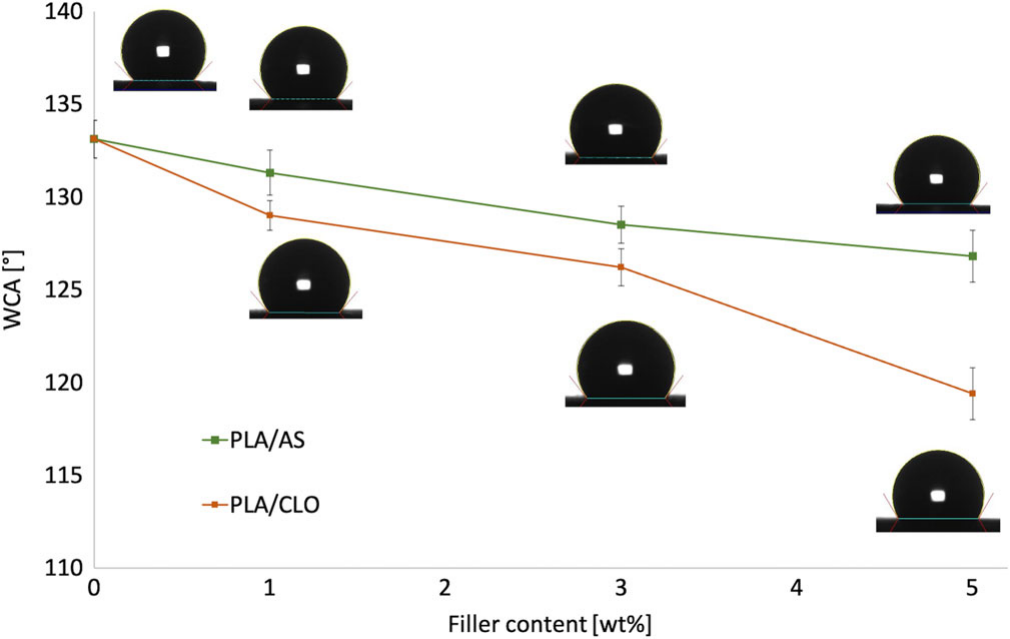
Water contact angles of the electrospun nanofiber mats. Values are given as means ± *SD* of *n* = 7 samples

The surface wettability of the bionanocomposites electrospun mats was analyzed to evaluate the hydrophilic/hydrophobic character of the scaffolds through water contact angle (WCA) measurements (Figure 7). Electrospun PLA showed intrinsic poor hydrophilicity displaying a WCA value around 133° while the addition of both CLO and AS induced a slight decrease of this value upon increasing the number of particles filled in the polymer matrix. More in detail, PLA/CLO composite nanofiber mats showed a more pronounced WCA decrease than PLA/AS systems, down to 119.4° for PLA/CLO 5%. On the other hand, the lowest WCA displayed for the nanocomposites containing AS was 126.8° for PLA/AS 5% mat.

### 3.3 Thermal and mechanical properties of electrospun mats

**FIGURE 8:**
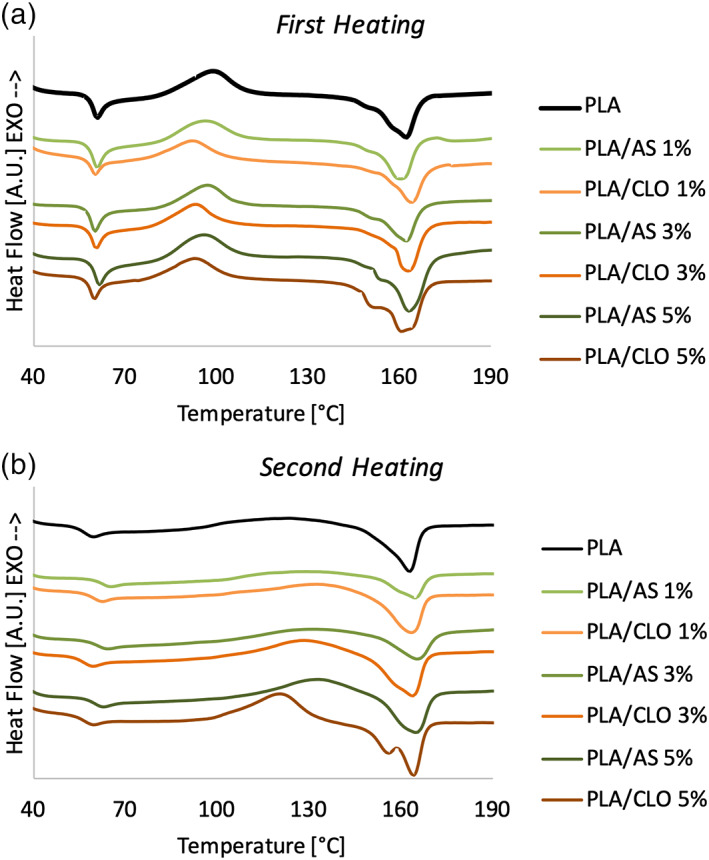
Differential scanning calorimetry (DSC) thermograms recorded during (a) first heating and (b) second heating of neat polylactic acid (PLA) and composites

DSC thermograms for the first heating and second heating scan for each non‐woven electrospun mat of the different PLA/AS and PLA/CLO formulations are reported in Figure 8a,b respectively, while their main thermal properties are summarized in Table 1.

**TABLE 1:** DSC results of electrospun PLA and composites

Samples	First heating					
	T_g_ (°C)	T_cc_ (°C)	T_m_ (°C)	ΔH_cc_ (J/g)	ΔH_m_ (J/g)	χ (%)
PLA	61.5	100.1	162.4	14.83	23.65	9.41
PLA/AS 1%	61.3	96.6	162.1	13.94	22.74	9.39
PLA/AS 3%	61.7	97.0	162.6	13.42	22.56	9.75
PLA/AS 5%	62.3	96.1	163.0	18.34	35.68	18.51
PLA/CLO 1%	60.8	92.9	163.7	9.15	19.30	10.83
PLA/CLO 3%	61.1	93.1	162.2	14.91	25.56	11.37
PLA/CLO 5%	60.2	93.5	164.9	11.40	35.25	25.45

Samples	Second heating					
	T_g_ (°C)	T_cc_ (°C)	T_m_ (°C)	ΔH_cc_ (J/g)	ΔH_m_ (J/g)	χ (%)
PLA	59.6	123.4	162.6	5.11	16.87	12.55
PLA/AS 1%	64.2	128.8	164.8	5.23	16.43	11.95
PLA/AS 3%	63.2	130.6	165.5	7.85	20.15	13.13
PLA/AS 5%	60.1	133.8	165.5	13.12	34.06	22.35
PLA/CLO 1%	62.6	130.9	163.2	8.38	22.00	14.54
PLA/CLO 3%	59.7	128.8	163.8	10.00	26.45	17.56
PLA/CLO 5%	59.5	120.1	164.0	14.76	34.56	21.13

Abbreviations: AS, silica; CLO, clay; DSC, differential scanning calorimetry; PLA, polylactic acid.

Electrospun PLA showed the *T*_g_‐related endothermic peak during the first and second heating scan at 61.5 and 59.6°C, respectively. The *T*_g_ of PLA seemed to be slightly affected by the presence of both AS and CLO in the first and in the second heating. The highest increase of T_g_ was observed during the second heating scan for the PLA‐based composites filled with the lowest amount of both AS and CLO.

Cold crystallization peaks were observed for both neat electrospun PLA and its composites. More in detail, cold crystallization temperature (*T*_cc_) decreased by about 3.5°C with the addition of AS and about 7°C with the addition of CLO and it seemed to be poorly affected by the amount of filler.

Neat PLA and composites showed double melting peaks being the dominant peak at the highest temperature around 162–164°C. Several researchers have observed similar melting behavior for PLA and its composites.^72^, ^73^ During the second heating scan, the double melting peak can be observed only for PLA/CLO 3 and 5%.

Results in Table 1 highlighted that during the first heating scan, the crystallinity of electrospun PLA was 9.41% and it remained almost constant for the composites containing 1 and 3% of AS while it increased up to 18.5% for PLA/AS 5% systems. Similarly, in PLA/CLO mats, PLA crystallinity slightly increased for PLA/CLO 1 and 3% while a strong χ increase (up to 25.4%) was observed for the composites containing the highest amount of the CLO.

During the second heating scan, the crystallinity behavior as a function of the kind and amount of filler was similar to that observed for the first heating scan. More in detail, χ of PLA/AS 1 and 3% remained almost similar to that of PLA (in the range 12–13%) while it increased up to 14.5 and 17.6% for PLA/CLO 1 and 3%, respectively. Similarly to the first heating scan, the highest increase of χ increase was observed for PLA/AS 5% and PLA/CLO 5% reaching the value 22.3 and 21.1%, respectively.

**FIGURE 9:**
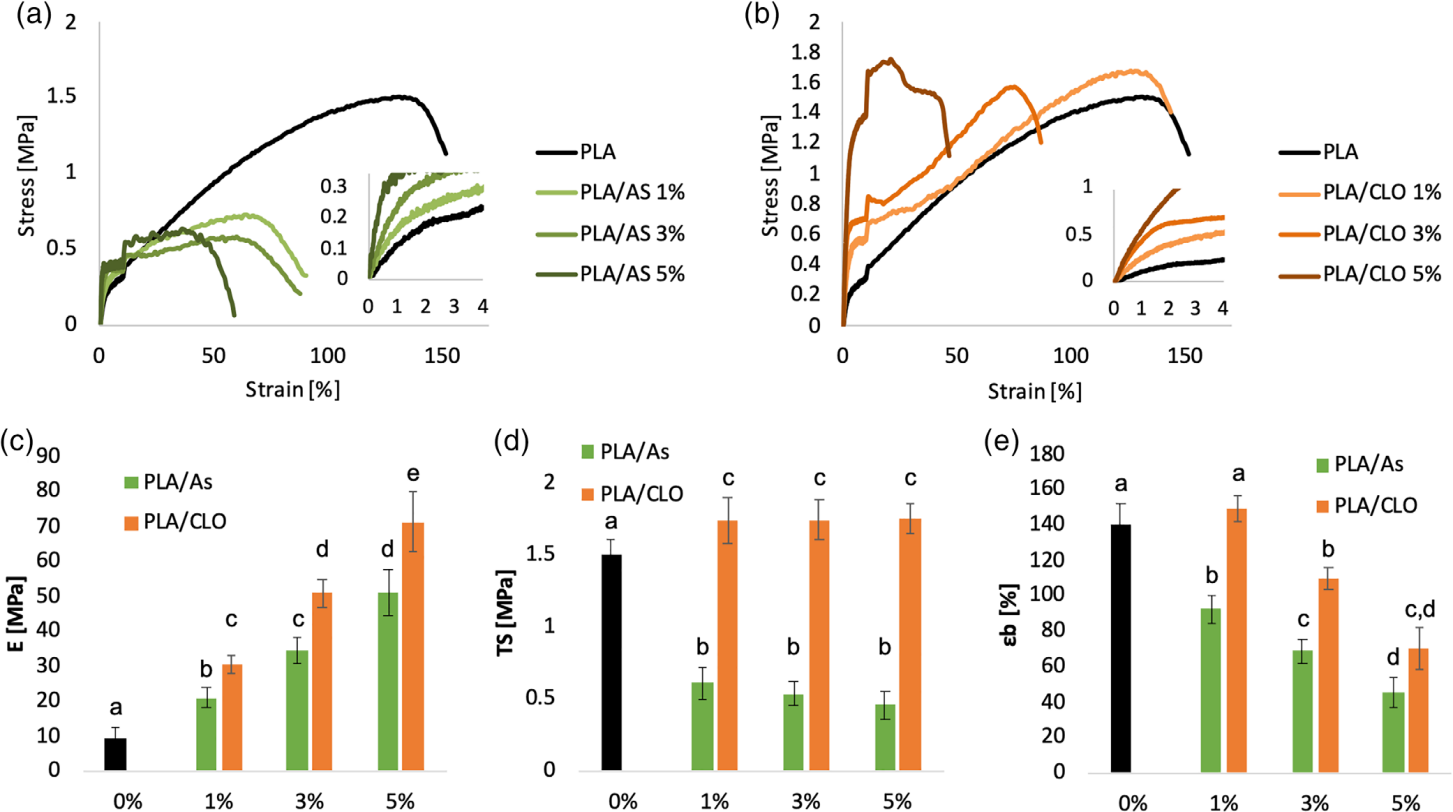
Representative stress–strain curves of (a) polylactic acid/silica (PLA/AS) and (b) PLA/clay (CLO) nancomposites electrospun mats; (c) elastic modulus, (d) tensile strength and (e) elongation at break of PLA/AS PLA/CLO nancomposites electrospun mats as a function of the filler content. Values are given as means ± *SD* of *n* = 5 samples. Different letters in the same graph indicate significant differences (*p* < .05) when analyzed by multiple Student's *t* test

Figure 9a,b represents the representative stress–strain curves of PLA/AS and PLA/CLO electrospun nanocomposites mats, respectively. The cusp points at 10% strain are due to the change of the crosshead speed. PLA electrospun mats showed the typical mechanical behavior of a ductile material exhibiting relatively low E and a relatively high ε_b_.

All PLA/AS composites were found to be more brittle than PLA as highlighted by the strong reduction of the elongation at break. On the other hand, PLA/CLO 1%, and partially also PLA/CLO 3%, mats showed a ductile behavior similar to that of electrospun PLA mats while only PLA/CLO 5% systems displayed the most brittle behavior among the PLA/CLO nanocomposites. AS reduced the tensile strength of the mats while for PLA/CLO systems it is possible to observe a slight increase of this parameter if compared to electrospun PLA.

In Figure 9c‐e the elastic modulus (E), the tensile strength (TS), and the elongation obtained from the stress–strain curves for both PLA/AS and PLA/CLO nanocomposites mats are reported as a function of the filler content, respectively. The data put into evidence that the elastic modulus of the membranes filled with AS and CLO (Figure 9c) increased almost linearly with the filler wt%. More in detail, comparing the composites with the same filler concentration, PLA/CLO elastic modulus increment was higher than that of PLA/AS and this difference was more pronounced upon increasing the amount of filler. In fact, E value of PLA/AS 1, 3, and 5% were 118, 278, and 438% higher than that of electrospun PLA, respectively, while the elastic modulus of PLA/CLO 1, 3, and 5% were 222, 438, and 653% higher than that of PLA, respectively.

As qualitatively observed in the stress–strain curves, the tensile strength values were strongly affected by the kind of nanoparticles filled in the polymer matrix. In particular, TS of PLA/CLO systems increased from 1.5 to 1.75 MPa with the addition of 1% of AS, then it remained almost constant for higher CLO concentration. On the other hand, the addition of 1% of AS induced a steep decrease of TS down to 0.6 MPa, and then it linearly decreased down to 0.46 MPa for PLA/AS 5% mats.

The elongation at break of PLA/AS nanocomposites mats significantly decreased if compared with electrospun PLA since from the addition of 1% of AS from 140 to 92% (Figure 9e). Then it linearly decreased upon increasing the AS amount down to 45% for PLA/AS 5% nanocomposites. On the other hand, the addition of the smallest amount of CLO here investigated did not significantly modify the elongation at break of PLA/CLO 1%. At higher CLO concentration the deformation decreased proportionally with the CLO content in the polymer matrix even if it was found to be always higher than that of PLA/AS systems.

### 3.4 Cell culture

The composite scaffolds cytocompatibility was assessed by in vitro cell proliferation of MC3T3‐E1 pre‐osteoblastic cells. For this study PLA, PLA/AS 1% and 5%, and PLA/CLO 1% and 5% samples were chosen.

**FIGURE 10:**
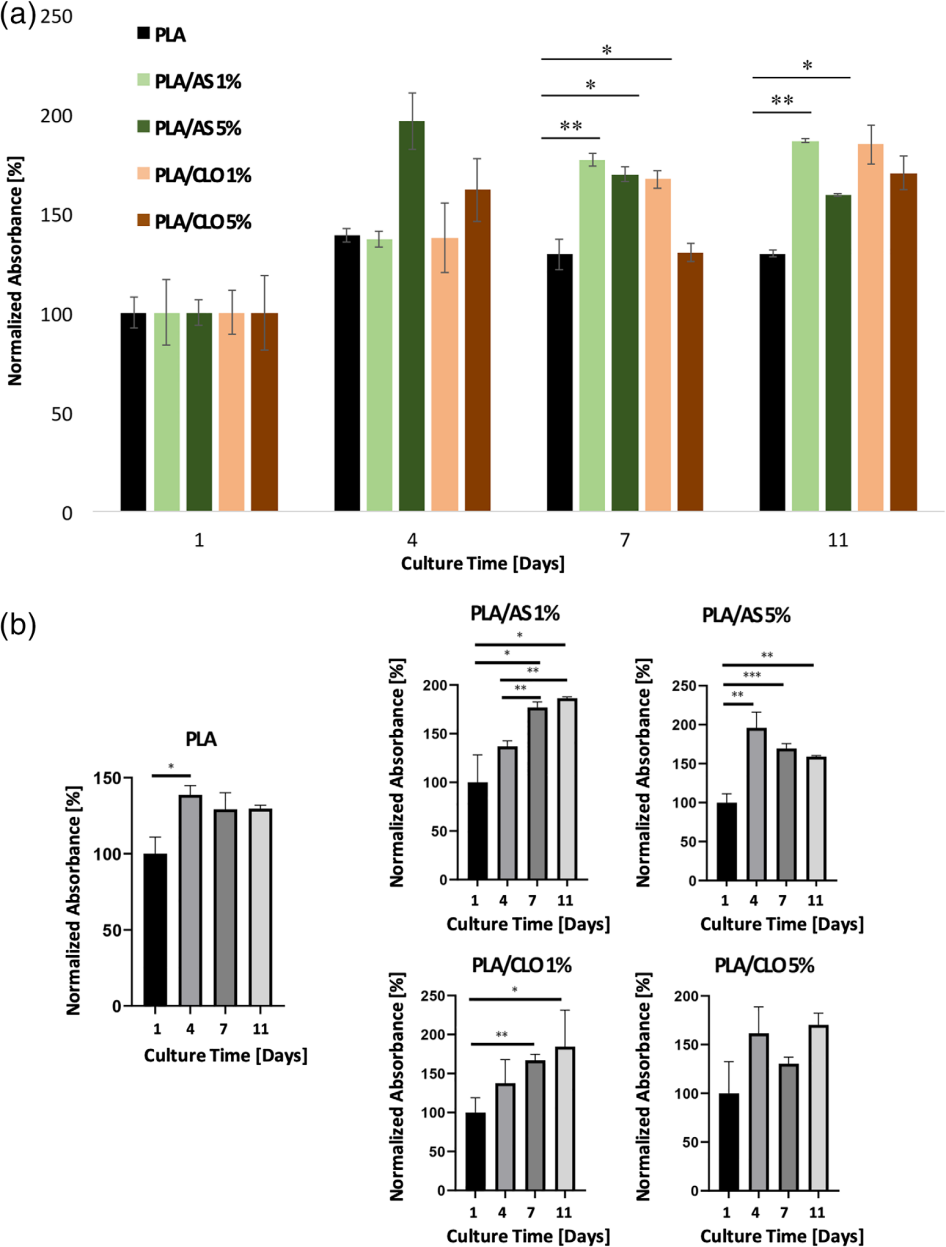
Viability assay carried out on cell grown at different times on polylactic acid (PLA), PLA/silica (AS) 1%, PLA/AS 5%, PLA/clay (CLO) 1% and PLA/CLO 5%. Values are given as means +*SD* of *n* = 3 samples. *p < .05; **p < .01; ***p < .001 when analyzed by Student's *t* test

Alamar assay was carried out to study the viability of cells seeded onto the different scaffolds. In order to obtain a reliable comparison, the absorbance values for each kind of sample were normalized with respect to the values obtained on theirs Day 1. From the graph in Figure 10a, it is possible to observe that at each time point, viable cell number in composite scaffolds is higher or at least equal with respect to PLA, chosen as control, confirming the non‐cytotoxicity of the employed materials and indicating a higher growth rate, probably due to the presence of the particles. In order to better appreciate the differences in terms of growth rate among the different samples, the same data were grouped for scaffold typology as a function of culture time, as shown in Figure 10b. It is interesting to highlight that in PLA and in 5% filled scaffolds a plateau is early reached from Day 4, whereas a statistically significant growth trend is noticeable for the 1% composite scaffolds (Figure 10b).

**FIGURE 11:**
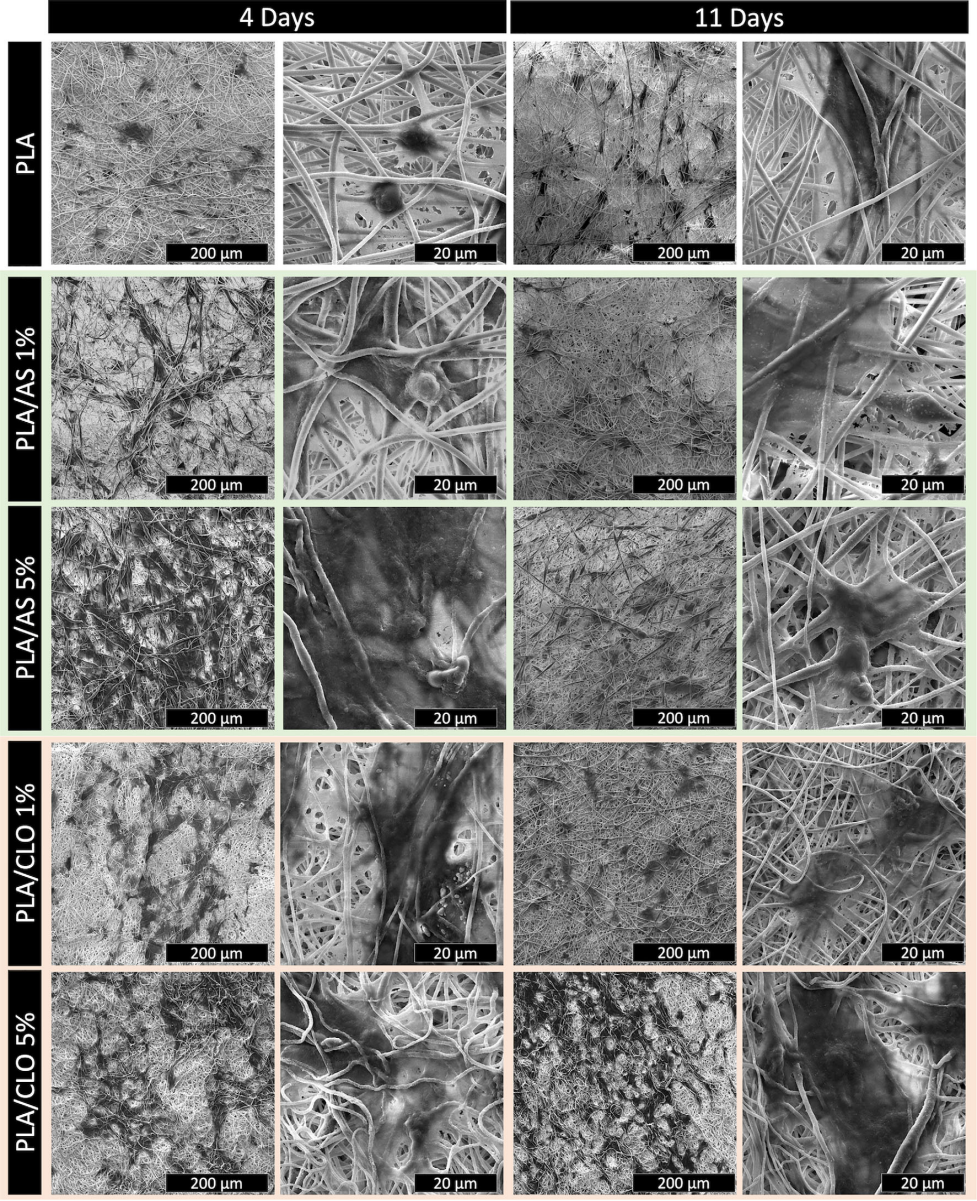
Scanning electron microscopy (SEM) micrographs at lower and higher magnification of pre‐osteoblastic cells grown on polylactic acid (PLA), PLA/silica (AS) 1%, PLA/AS 5%, PLA/clay (CLO) 1% and PLA/CLO 5% at different culture times

The distribution and morphology of the cells grown onto the scaffold are shown in Figure 11 for all the mats after 4 and 11 days of culture. From the SEM micrographs, it is possible to appreciate that pre‐osteoblastic cells cultured for 4 days on composite mats aggregated more abundantly if compared with PLA control that showed only isolate cells or, at least, little cell aggregations. More in detail, the number and size of aggregated cells increased when increasing the amount of AS or CLO particles into the polymer matrix. Furthermore, at higher magnification, SEM images highlighted that pre‐osteoblastic cells cultured on PLA/AS and PLA/CLO systems spread out more rapidly if compared with the electrospun PLA. In fact, after the fourth day of culture, cells on PLA mats exhibited a not‐spread round shape whereas the other systems exhibited a well‐spread cell morphology. After 11 days of culture, the only system showing a still well‐aggregated cell morphology is PLA/CLO 5%, although pre‐osteoblastic cells were found to be well‐spread in all the systems here investigated.

## 4 DISCUSSION

In this work, it was assessed the physical and biological properties of PLA electrospun bionanocomposites filled with two different commercial nanofillers that is, nanosilica (Aerosil® R 812) and nanoclay (Cloisite® 30B). Aerosil R812 has a silicon dioxide content of over 99.8% thus it is an excellent candidate for application in tissue engineering due to its chemical inertness that has no known health implications.^74^ Silica and its derivatives were introduced as bone substitutes^75^ demonstrating clinical success rates in terms of promotion of new vital bone and as a bio‐mimetic coating for implant surfaces.^52^, ^76^ In this context, silica nanoparticles can form a tighter interface with the polymer matrix in composites due to their large specific surface area then this filler can not only endow polymer scaffolds with biomineralization capability but also increases the stiffness of polymer materials.^77^ On the other hand, Cloisite 30B, an organically modified montmorillonite, is one of the most used nanoparticles because of its capacity to improve the mechanical and thermal properties of the polymer matrix.^78^ Furthermore, the quaternary ammonium moiety present in Cloisite 30B has been shown to have antimicrobial properties.^79^

SEM and BET analysis conducted on the fillers highlighted that AS particles are smaller than CLO. This feature can affect several physical and biological properties of the nanocomposite mats such as fiber diameter, roughness, and, as a consequence, its biocompatibility.

The FTIR‐ATR spectra confirmed the successful inclusion of the fillers in the PLA nanofibers. In fact, PLA/AS and PLA/CLO systems showed the characteristic peaks of both the polymer matrix and the respective nanoparticles. Also, the presence of peaks at 2,925 and 2,853 cm^−1^ due to alkylammonium ions in Cloisite 30B in PLA/CLO nanofibrous composites reveals that the organo‐modifier in Cloisite 30B was still present after processing. The alkylammonium ions preservation after 1 hr sonication of CLO in TCM:Ac (2:1 vol) can be likely ascribed to the packed morphology of these particles. In fact, the organo‐modifier is intercalated among the MMT layers that, as clearly visible from SEM and STEM images, maintained their stuck structure also after the electrospinning process thus preserving the salt from dispersion in the solvent system.^70^

The fiber morphology and diameter of PLA‐based electrospun scaffolds were affected by both the filler weight concentration and type, and both features are furtherly related to the solution viscosity. More in detail, the PLA/AS fiber diameter was found to be higher than that of neat electrospun PLA. These results can be likely explained by considering that the electrospinning process is based on the uniaxial stretching of a charged droplet of polymeric solution. The stretching degree can be strongly affected by the polymeric solution/suspension viscosity since the higher the shear viscosity, the higher the resistance to flow. For this reason, the electrospun fiber diameter increase upon increasing the solution viscosity, as already observed in other works.^26^, ^80^, ^81^, ^82^ It is well known that solid particles can increase the polymeric suspensions complex viscosity except for nanoparticles presenting pro‐degradative effects or acting as plasticizers.^83^, ^84^, ^85^ In this work, the introduction of AS induced an increase of the complex viscosity in the polymer solution that can explain the increase of the electrospun fiber diameter.

The spread diameter size distribution of the PLA/AS system can be related to the presence of AS agglomerates observed by STEM analysis but also to the increased viscosity, which was found to be more accentuated at the low frequencies. This feature can induce instability of the jet near the Taylor cone and, as a consequence, to the not‐homogenous fiber size distribution observed by SEM micrographs. The different viscosity observed at the low frequencies for the PLA/particles suspensions can be related to the dimension of the fillers. In fact, AS particles were found to be smaller than that of CLO thus exhibiting higher specific surface area and, as a consequence, a higher capacity to create a 3D network in the suspension than CLO particles.

At the same time, CLO particles caused a decrease of the PLA fiber diameter as already observed previously for different polymer matrices^43^, ^54^ and a more homogenous diameter size distribution. The fiber size reduction can be likely ascribed to the conductivity of the PLA solution that can strongly affect the morphology and diameter of the electrospun nanofibers. As already observed, CLO particles contain a quaternary ammonium ion as an organic modifier and Na^+^ and Ca^2+^ ions located between MMT layers. This organo‐modifier can likely augment the conductivity of PLA/CLO dispersions upon increasing the amount of CLO added, as already observed for other polymeric suspensions^44^ and, as a consequence, induce the significant decrease in the average diameter observed for PLA/CLO nanofibers.

Differently from PLA/AS systems, the more homogenous diameter distribution of PLA/CLO fibers can be related to the viscosity of the PLA/CLO suspensions at the low frequency that is very near to that of PLA solution thus avoiding the instability of the Taylor cone. Furthermore, CLO particle size in PLA/CLO mats is lower than that observed for sonicated neat CLO particles. One of the reasons for achieving this result may be likely ascribed to the strong elongational force applied to the solution during the electrospinning process, which can induce the rupture of CLO particles during the nanofibers formation^54^ although no exfoliation phenomena between CLO layers were observed by STEM investigation.

The WCA measurements revealed that both the nanofillers cause a slight increase of the scaffolds wettability and that it increased upon increasing the filler loading. In order to explain these results, it may be taken into account that the wettability performance of nanocomposites is strongly dependent on the surface topographical properties but also on the chemical properties of the filler.^86^ Although the increased diameter of the PLA/AS fibers can induce an increase of the WCA by reducing the specific surface of the mat, the slight increase in hydrophilicity observed for the PLA/AS systems can be ascribed to the presence of silicon dioxide on the nanosilica surface.^87^ On the other hand, the more pronounced WCA reduction observed for PLA/CLO systems can be likely ascribed to the thinner fiber diameter observed by SEM and to the presence of the organo‐modified clays at the surface of the fibers, as highlighted by FTIR‐ATR measurements.^88^, ^89^

Electrospun PLA usually exhibits much lower crystallinity than that of PLA in pellets because of the high speed of solidification during the process.^90^ During the jet, the PLA chains are not able to crystallize because of the rapid solidification due to the solvent evaporation, therefore, the electrospun PLA specimens were nearly amorphous (χ_PLA_ = 9.41%). DSC thermograms also highlighted that PLA melting behavior is dependent on the kind and concentration of particles filled in the matrix. More in detail, from the data recorded during the first heating scan it was possible to conclude that AS increased the PLA crystallinity only at the highest concentration (χ_PLA/AS 5%_ = 18.51%) while CLO induced a slight crystallinity increase also at lower concentration and steep growth of this parameter up to 25.45% for PLA/CLO 5% nanocomposites. Based on the similar crystallinity increment observable also during the second heating scan, it can be assumed that both the fillers acted as a nucleating agent for PLA. Moreover, it can be highlighted that PLA/CLO particles showed a higher crystallinity than PLA/AS bionanocomposites.

From a mechanical point of view, both AS and CLO particles were able to increase the elastic modulus of the nanofibrous mats with differences related to the filler kind and amount. Specifically, the elastic modulus of PLA/CLO 1%, 3%, and 5% is 48, 42, and 40% higher than that of the corresponding PLA/AS systems. Much more evident are the differences among TS values of the two nanocomposites. Differently from PLA/AS mats, PLA/CLO systems exhibited TS higher than PLA. This result can be explained by observing the stress–strain curves in Figure 9a,b and the elongation data in Figure 9e. These results highlight that PLA/AS composites are less deformable than PLA and PLA/CLO mats, that is, the failure occurred prematurely for PLA/AS systems thus inducing smaller TS values for these composites. In the case of PLA/CLO mats, the premature failure of the samples is compensated by the higher increase of the elastic modulus that, as a result, led to TS values slightly higher than that of PLA.

These results can be ascribed to several features of PLA/CLO and PLA/AS electrospun nanocomposites including (a) fiber morphology, (b) crystallinity, and (c) affinity between PLA and fillers. More in detail, PLA/CLO fibers were characterized by more homogenous diameter size distribution and by smaller mean diameter than PLA/AS composites. Both these features usually lead to mats exhibiting higher elastic modulus and higher deformation at break.^91^, ^92^ In particular, the presence of the bead‐like irregularities in PLA/AS fibers can act as defects of the structures thus easing the premature fracture of the mats.^18^

The different crystallinity of electrospun PLA and its composites can be considered another element able to explain the higher elastic modulus of PLA/CLO mats if compared with neat electrospun PLA and PLA/AS, although it is not coherent with the lower deformation at break of PLA/AS systems that is probably more ascribable to their fiber morphology. Finally, another potential key factor that can likely explain the different mechanical behavior of PLA/AS and PLA/CLO can be ascribed to the different affinity between PLA and the fillers. In fact, from these results, it can be supposed a better affinity between PLA and CLO than PLA and AS, likely due to the presence of the organo‐modifier in the MMT nanoparticles.

Biological in vitro tests confirmed the biocompatibility of all proposed devices. Preliminary data seem to indicate that the presence of the filler induces a positive effect on the proliferation and adhesion of the pre‐osteoblastic cells.

The different growth trends of MC3T3‐E1 pre‐osteoblastic cells on different scaffolds can be ascribed to several factors related to intrinsic and extrinsic properties of the nanoparticles filled in the polymer matrix including specific surface areas, surface charge, functionality, size as well as the improvement of the mechanical properties and wettability of the scaffolds that play direct roles in determining specific cellular responses.^93^ At lower filler concentration (i.e., PLA/AS 1% and PLA/CLO 1%) and in the first time point (i.e., 4 days) all these features can likely explain the significant growth trend observed in our systems.

On the other hand, the slight decrease in cell growth observed on PLA/AS 5% starting from Day 4 can be likely ascribed to potential metabolic activity changes induced by AS particles. In fact, in other electrospun nanocomposite filled with nanosilica, it was observed that the higher the filler concentration the higher the number of nanoparticles that can adhere to the cell surface and then be internalized thus restricting the cellular functionality.^94^ Moreover, high concentration of silicate nanoparticles can also interact with the media proteins and results in the formation of aggregates that cannot be engulfed by the cells. This might also contribute to decreasing the growth trend at higher silicate concentrations.^94^

The effect of electrospun polycaprolactone filled by clay nanoparticles on protein absorption was investigated by Gaharwar et al.^55^ Their results highlighted the low protein adsorption induced by these particles. Furthermore, the relatively high dimension of CLO particles reduces the possibility to be internalized by cells. Therefore, the cell growth plateau observed in PLA/CLO 5% scaffolds after 4 days could be related to the release of the alkylammonium salt intercalated into the clays into the medium that can thus affect the cell growth. In fact, it is well known that the alkyl ammonium surfactant present in Cloisite 30B can be released in a liquid medium also when incorporated in polymer matrices and it is primarily responsible for its microbicidal properties.^38^

Coherently with viability assay results, SEM micrographs carried out on the electrospun mats after 4 days of culture revealed that upon increasing both AS or CLO particles filled into the polymer matrix, the number and size of clusters of aggregated cells increased.

The only system likely able to exhibit a biofilm‐like structure after 11 days of culture is PLA/CLO 5%. Also, the spread morphology of the cells cultured on the composites mats revealed that both AS and CLO particles were likely able to enhance the adhesion of the pre‐osteoblastic cells on the electrospun systems.

These results can be presumably ascribed to the enhanced elastic modulus and wettability of the electrospun mats filled with AS and CLO particles. These data suggest that both AS and CLO nanoparticles embedded in PLA electrospun matrix provided a favorable cell proliferation environment that can be ascribed to the increased cell affinity for the substrate. Further tests will be carried out in order to better understand the influence of these particles on cell proliferation and differentiation and will be reported in a separate paper since a detailed analysis goes far beyond the scope of the present paper.

## 5 CONCLUSIONS

In this work, the physical and biological properties of electrospun PLA, PLA/AS, and PLA/CLO bionanocomposites were evaluated.

The successful inclusion of both AS and CLO particles in PLA fibers was confirmed by SEM images and FTIR‐ATR that demonstrated also the preservation of the Cloisite 30B organo‐modifier.

The AS loading led to an increase of the mean fiber diameter and a less homogenous fiber size distribution of PLA/AS systems probably due to the high viscosity of the processing suspensions observed at the low frequencies. On the contrary, CLO induced a decrease of the mean fiber diameter and a more homogenous fiber size distribution likely ascribed to the presence of the quaternary ammonium salt in the MMT particles. The organo‐modifier of Cloisite 30B was also able to reduce the WCA of the PLA/CLO electrospun composites.

DSC analysis revealed that both AS and CLO nanoparticles increased the crystallinity of PLA in both the heating scans performed acting as a nucleating agent for the polymer matrix.

CLO reinforcing effect was higher than that of AS particles. In fact, PLA/AS scaffolds highlighted a more brittle behavior than electrospun PLA or PLA/CLO nanocomposites, probably because of the bead‐like structures formed in the fibers that acted as a local defect. The more intense increment of the elastic modulus due to CLO particles was related to the thinner fibers formed during the electrospinning, to the higher crystallinity of PLA/CLO composites, and their better affinity with the polymer matrix, likely due to the presence of the organo‐modifier.

Preliminary biological in vitro assays highlighted the biocompatibility of PLA/AS and PLA/CLO bionanocomposites.

The different growth trends of MC3T3‐E1 pre‐osteoblastic cells on different scaffolds can be likely ascribed to several features related to the different properties of CLO and AS nanoparticles. The increased mechanical properties and wettability of the scaffolds can likely explain the significant growth trend observed in nanocomposite systems at the lowest filler concentration and in the first time point. On the other hand, the low dimension of AS particles and the presence of alkyl ammonium additives on CLO could be responsible for the plateau on the growth trend after 4 days in the system at higher filler concentration.

Furthermore, both types of nanoparticles were able to induce a spread morphology of the cells cultured on the composites and to drove the cells toward uniform colonization onto the scaffolds. Based on these results, it is possible to identify AS and CLO as reliable candidates as fillers for electrospun PLA devices for bone tissue engineering applications.

## CONFLICT OF INTEREST

The authors declare no potential conflict of interest.

## AUTHOR CONTRIBUTION

Conceptualization: Francesco Lopresti and Vincenzo La Carrubba; Writing–original draft: Francesco Lopresti, Francesco Carfì Pavia; Writing–review and editing: Francesco Lopresti, Manuela Ceraulo, Elisa Capuana, Valerio Brucato, Giulio Ghersi, Luigi Botta, and Vincenzo La Carrubba.

## Data Availability

The data that support the findings of this study are available from the corresponding author upon reasonable request.
